# Supplementary figures and images for: Potential impact of annual vaccination with reformulated COVID-19 vaccines: Lessons from the US COVID-19 scenario modeling hub
Source: PLoS Med. 2024 Apr 17;21(4):e1004387. doi: 10.1371/journal.pmed.1004387 (PMC11062554; doi:10.1371/journal.pmed.1004387)

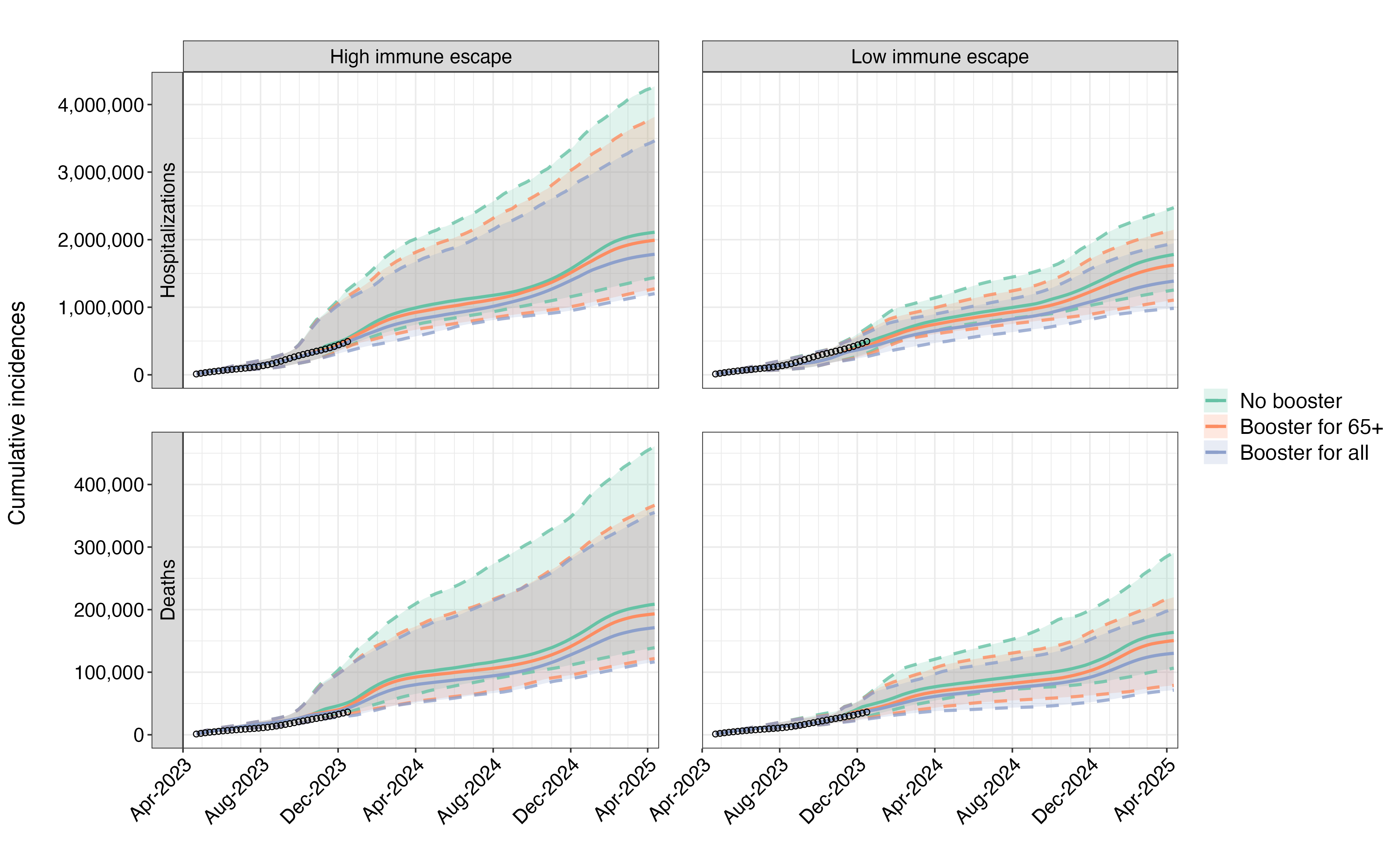

Supplement: S1 Fig — Ensemble projections for cumulative COVID-19 hospitalization and deaths in the United States for the next 2 years (April 2023–April 2025) are shown by scenario. Solid lines indicate the median of projected outcomes, and dash lines and shades indicate their 90% projection intervals. Each color represents different annual vaccination recommendations (no recommendation, reformulated vaccines recommended for those aged 65 and above, and recommended for all age groups). Dots indicate the observed cumulative hospitalizations and deaths from April 15, 2023 and December 16, 2023. (TIF) [file pmed.1004387.s001.tif]

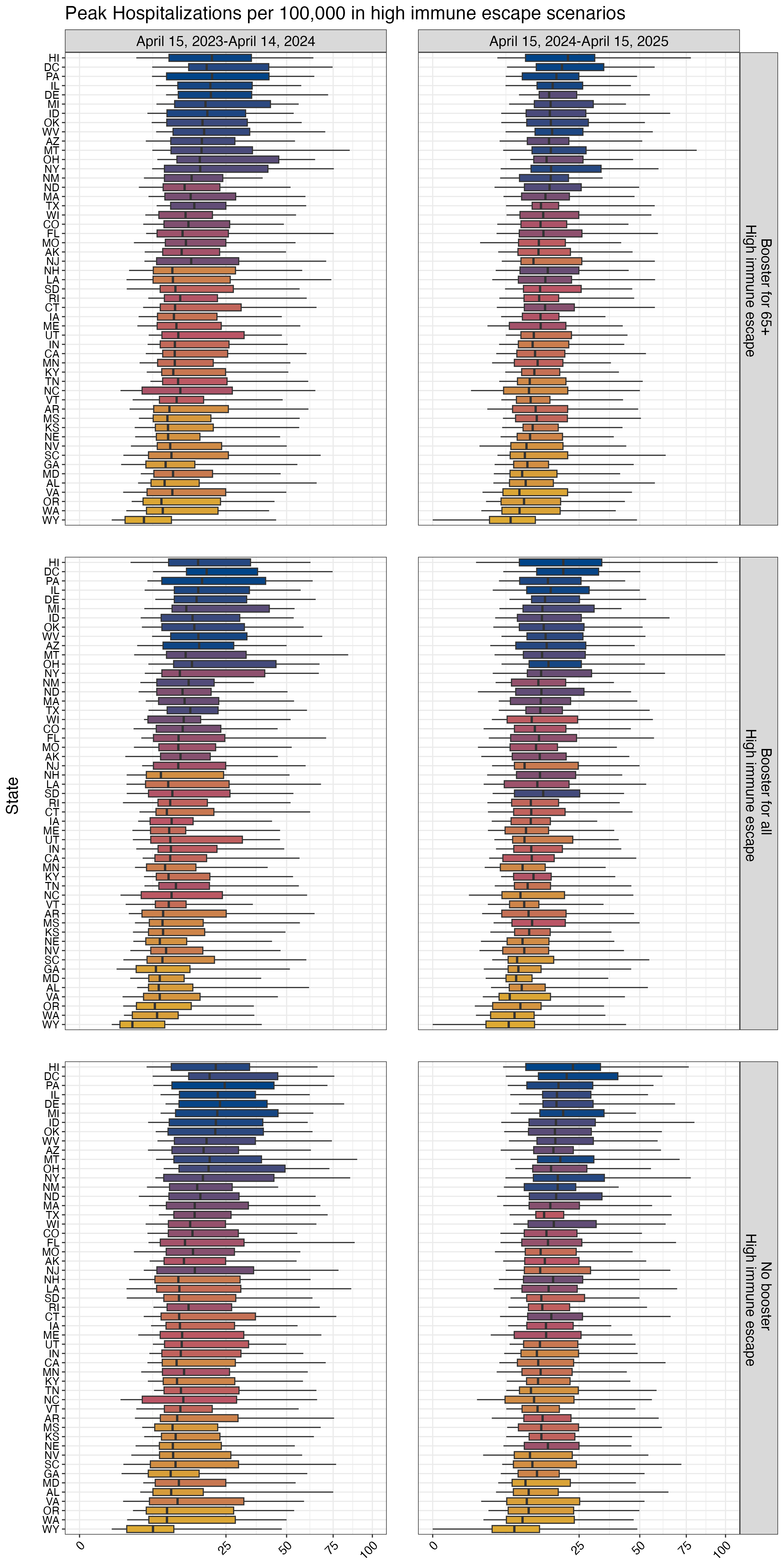

Supplement: S2 Fig — The peak hospitalizations per 100,000 over the next 2 years (April 2023–April 2025) under high immune escape assumption are shown by US state and by vaccination scenario (no recommendation, reformulated vaccines recommended for those aged 65 and above, and recommended for all age groups). The color variation denotes the order of US states in the peak hospitalizations by scenario and season. Shades of yellow indicate states with lower values and shades of blue indicate states with higher values. For visualizations, square root scaling was applied in x-axes. (TIF) [file pmed.1004387.s002.tif]

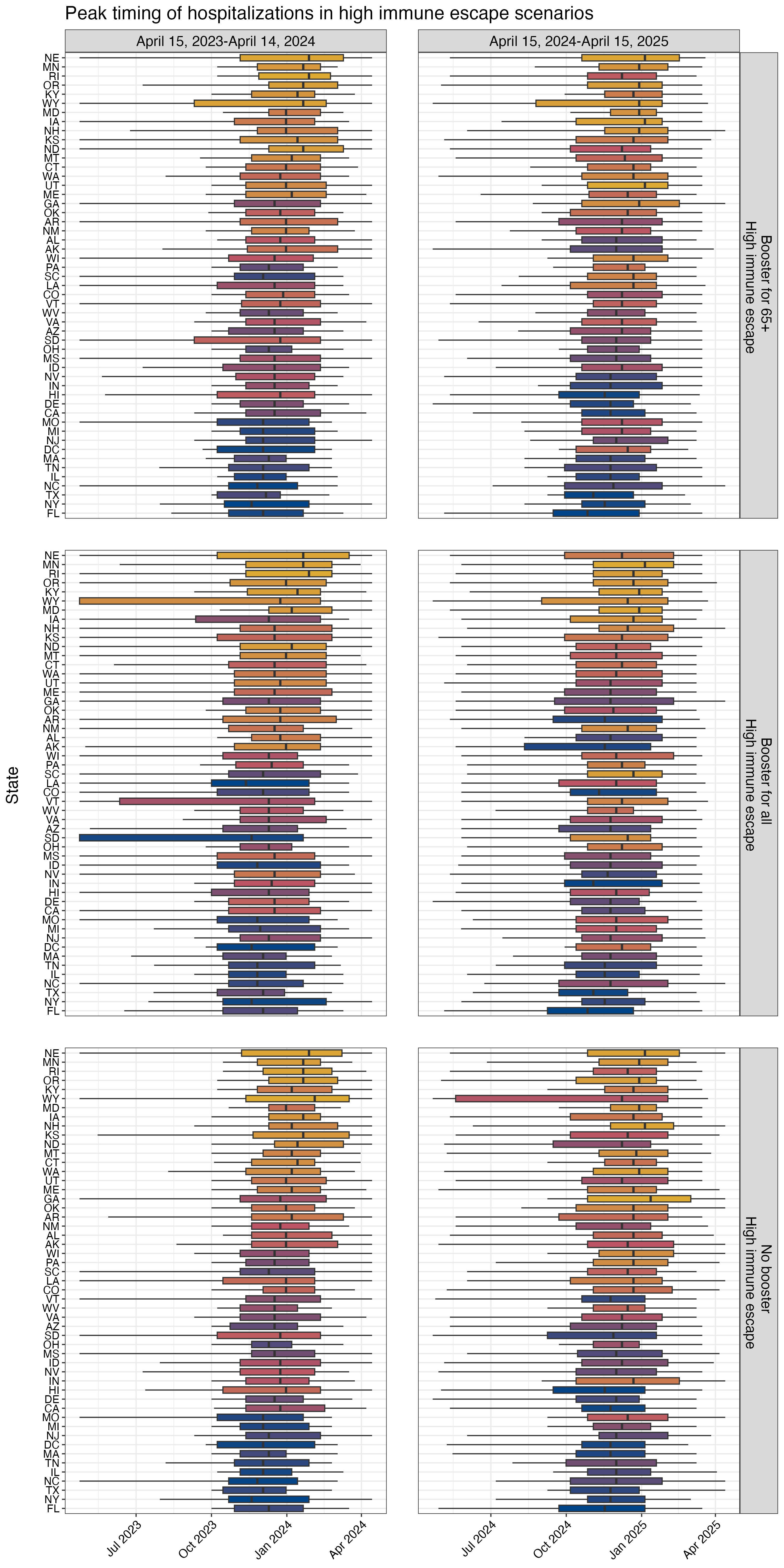

Supplement: S3 Fig — The peak timing of hospitalizations under high immune escape assumption is shown by US state and by vaccination scenario (no recommendation, reformulated vaccines recommended for those aged 65 and above, and recommended for all age groups). The color variation denotes the order of US states in the peak timing of COVID-19 hospitalizations by scenario and season. Shades of blue indicate states with an earlier peak and shades of yellow indicate states with a later peak. (TIF) [file pmed.1004387.s003.tif]

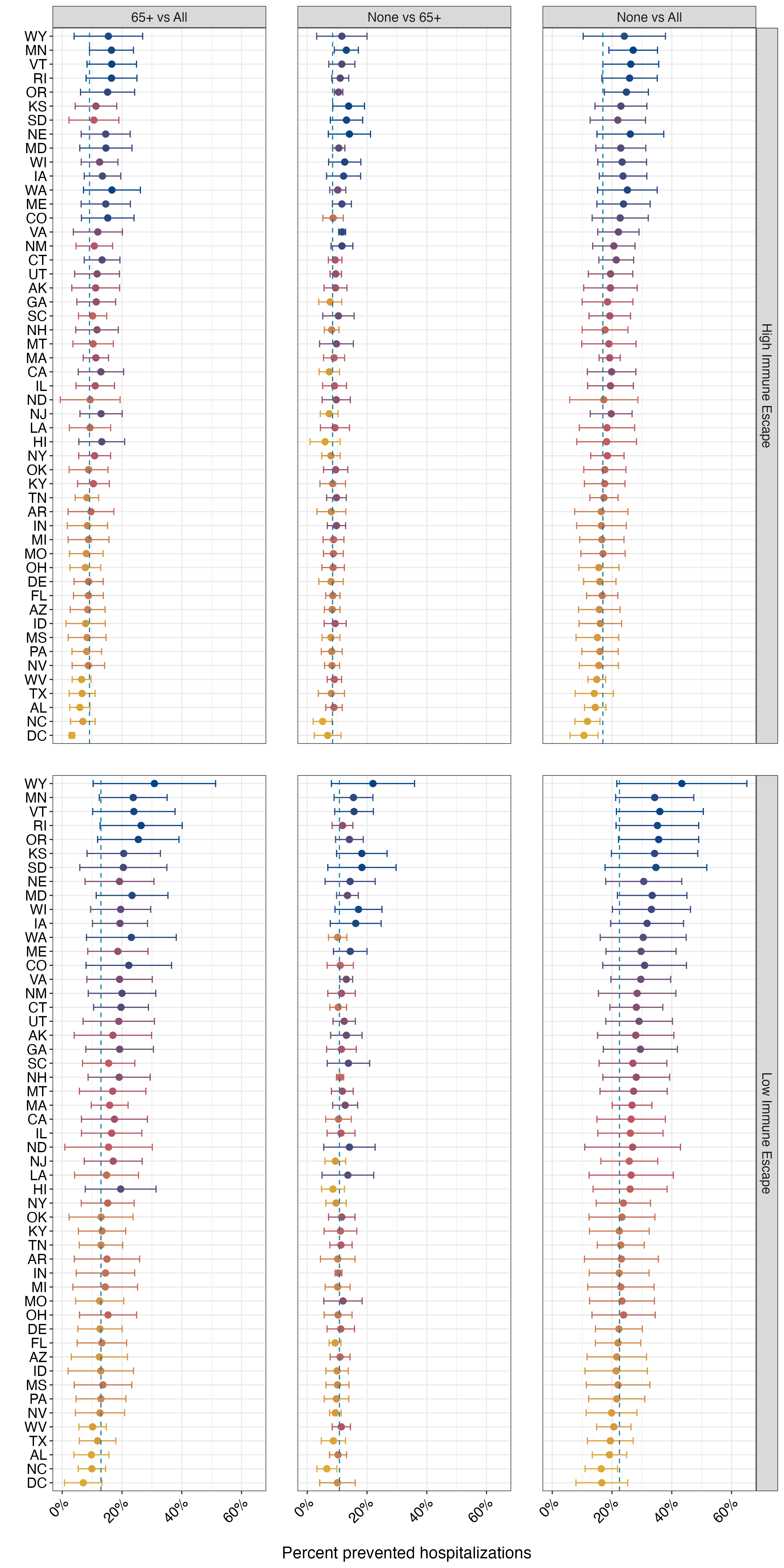

Supplement: S4 Fig — Relative differences in cumulative COVID-19 hospitalizations over the next 2 years (April 2023–April 2025) between different vaccination scenarios are shown by immune escape level and by US state. The color variation denotes the order of US states in the percent prevented hospitalizations by scenario. Shades of yellow indicate states with lower values and shades of blue indicate states with higher values. (TIF) [file pmed.1004387.s004.tif]

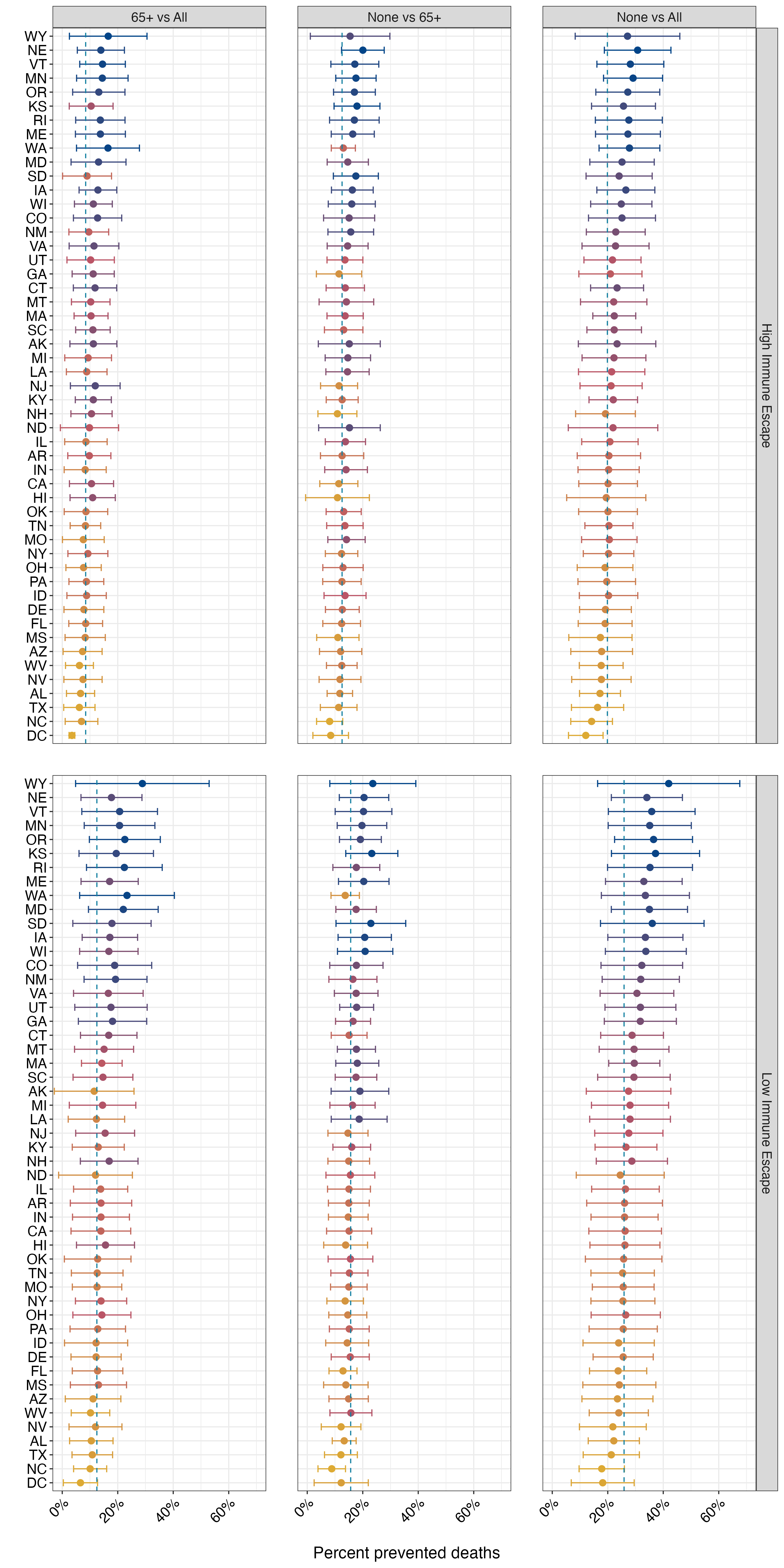

Supplement: S5 Fig — Relative differences in cumulative COVID-19 deaths over the next 2 years (April 2023–April 2025) between different vaccination scenarios are shown by immune escape level and by US state. The color variation denotes the order of US states in the percent prevented deaths by scenario. Shades of yellow indicate states with lower values and shades of blue indicate states with higher values. (TIF) [file pmed.1004387.s005.tif]

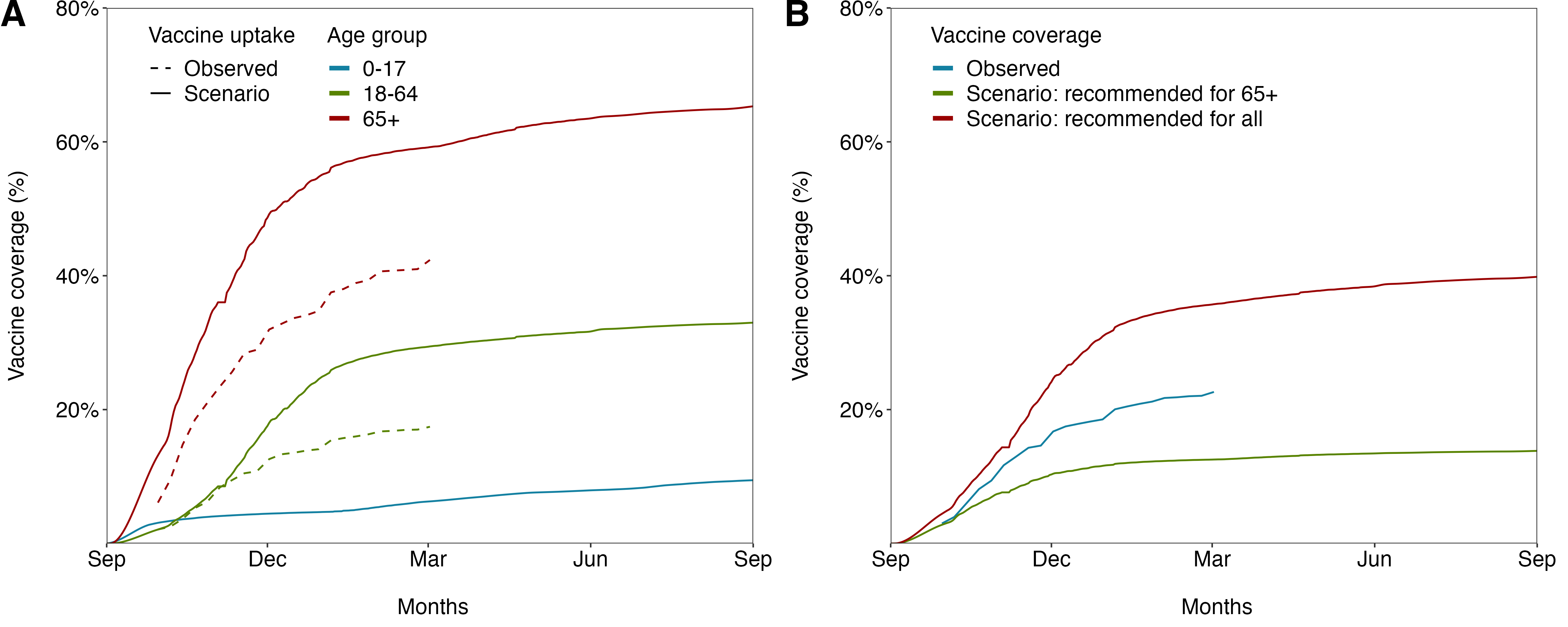

Supplement: S6 Fig — (A) Solid lines represent the assumed national-level annual uptake of reformulated vaccines by age group, projected to follow the uptake patterns for the first booster dose (authorized in September 2021). Dashed lines indicate the empirically observed uptake as of February 24, 2024, sourced from the CDC website, covering Puerto Rico and the Virgin Islands which are not accounted for in the assumed national-level uptake. Each age group is represented by a different color. (B) Observed and assumed annual uptake of reformulated vaccines among individuals aged 18 and over at the national level. Each color represents a different vaccine coverage data. (TIF) [file pmed.1004387.s006.tif]

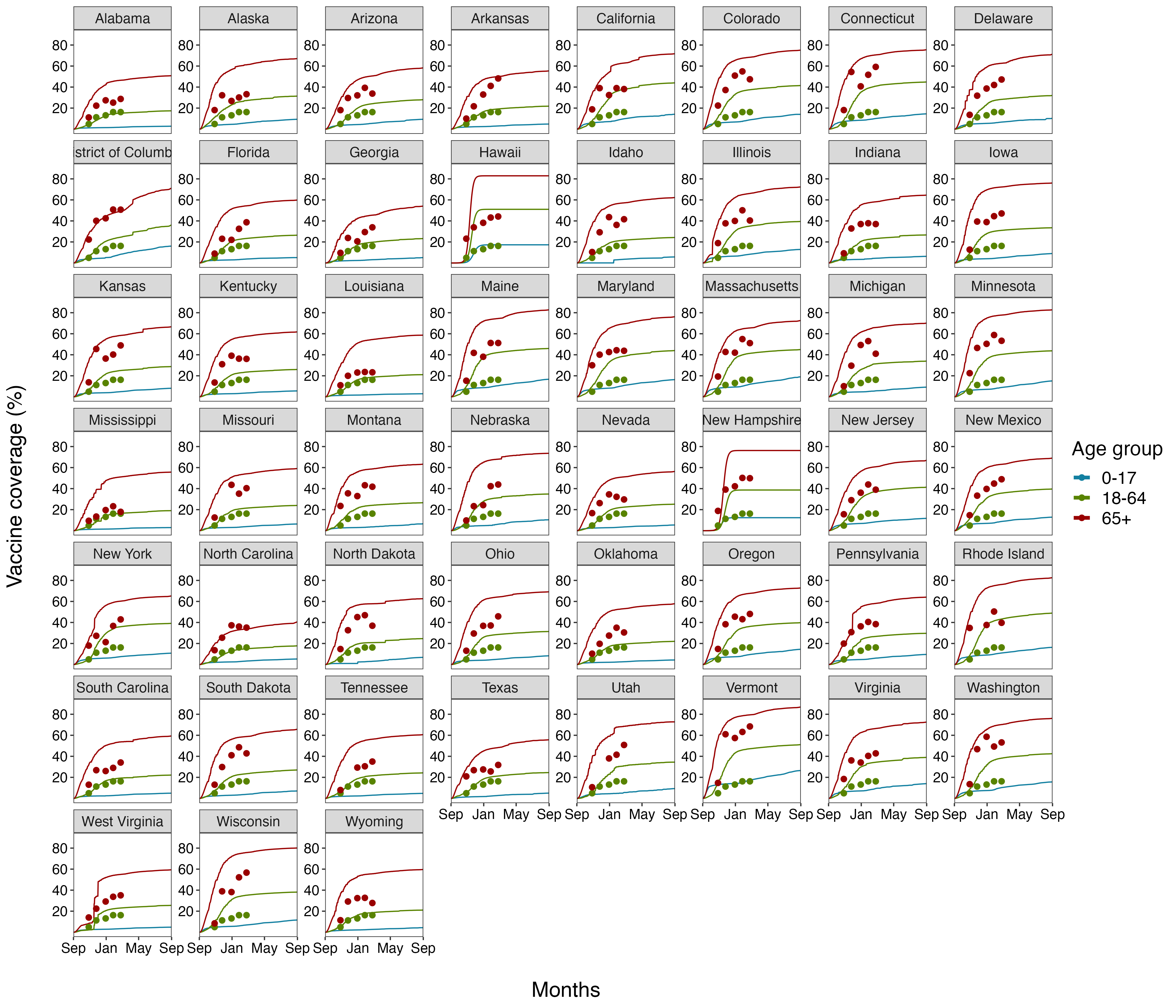

Supplement: S7 Fig — Solid lines represent the assumed state-level annual uptake of reformulated vaccines by age group, projected to follow the uptake patterns for the first booster dose (authorized in September 2021). Dots indicate the monthly observed uptake as of February 24, 2024, sourced from the CDC website. Each age group is represented by a different color. (TIF) [file pmed.1004387.s007.tif]

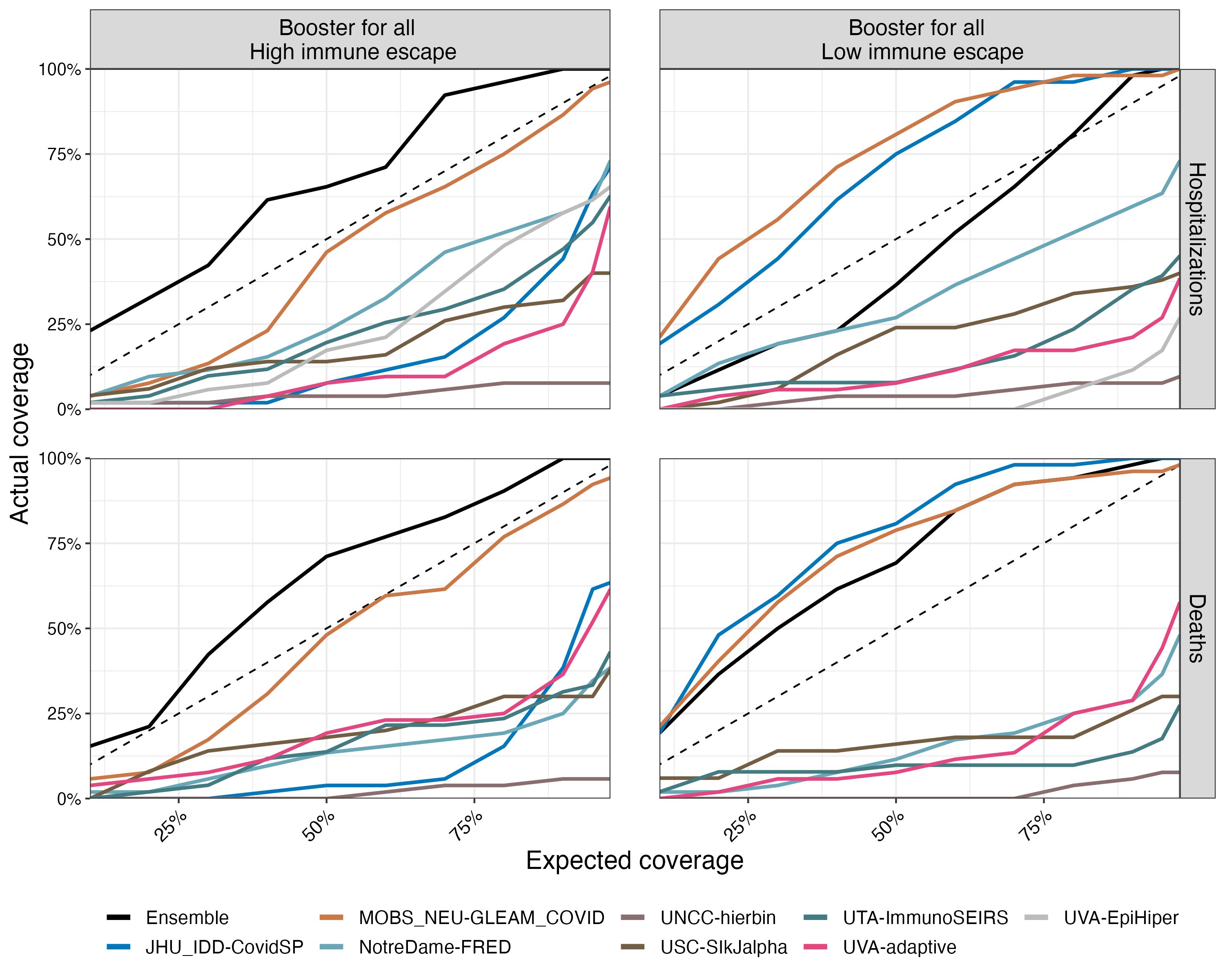

Supplement: S8 Fig — The actual coverage of each model, regarding cumulative hospitalizations and deaths as of December 16, 2023, is plotted against its expected coverage. Coverage measures the percentage of observations that fall within a given prediction interval (e.g., for a 90% prediction interval, expected coverage is 90%). Coverage was calculated across all locations and projection weeks. The dashed lines represent the expected relationship (expected coverage is equal to actual coverage), where a line below indicates models are overconfident (actual coverage is less than expected coverage), and above the line means models are underconfident (actual coverage is more than expected coverage). The black solid lines depict the ensemble model, while each colored line represents contributing individual models. Following the CDC recommendation for reformulated vaccines (published on September 12, 2023), only scenarios with vaccination recommendations to all individuals were included. (TIF) [file pmed.1004387.s008.tif]
