# Supplementary material for: Potential impact of annual vaccination with reformulated COVID-19 vaccines: Lessons from the US COVID-19 scenario modeling hub
Source: PLoS Med. 2024 Apr 17;21(4):e1004387. doi: 10.1371/journal.pmed.1004387 (PMC11062554; doi:10.1371/journal.pmed.1004387)
Supplement: S1 Table — (DOCX) [file pmed.1004387.s009.docx]

**S1 Table. Detailed description of individual models**

|  | **JHU_IDD-flepiMoP** [1] | **MOBS-NEU-GLEAM_COVID** [2] | **NotreDame-FRED** [3] | **UNCC-hierbin** | **USC-SIkJalpha** | **UTA-ImmunoSERIS**  [4] | **UVA-adaptive** | **UVA-EpiHiper** | **NCSU-COVSIM**  [5] |
| --- | --- | --- | --- | --- | --- | --- | --- | --- | --- |
| **Model type** | Meta-population compartmental | Meta-population compartmental | Agent-based | Non-mechanistic deep learning | Compartmental | Meta-population compartmental | Meta-population compartmental | Agent-based | Agent-based |
| **Geography** | State level | County level | State level | State level | State level | State level | State level | Individual level | North Carolina |
| **Age structure (age groups)** | 3 (0-17, 18-54, 65+) | 10 (0-9, 10-19, 20-24, 25-29, 30-39, 40-49, 50-59, 60-69, 70-79, 80+) | Individual ages | None | 5 (0-4, 5-11, 12-17, 18-64, 65+) | 6 (0-4, 5-11, 12-18, 19-49, 50-64, 65+) | None | Individual ages | 5 (0-4, 5-9, 10-19, 20-64, 65+) |
| **Other demographic structure** | None | None | None | None | None | None | None | None | Individual with diabetes, race/ethncity |
| **Mobility & Contact patterns** | Commuting | Google mobility, commuting, flight, age contact matrices | Google mobility | None | Cubiq contact index | None | None | Commuting, Natonal Household Travel survey | Contacts based on Census data |
| **Annual antigenic drift** | Linear | Linear | Linear | Linear | Linear | Linear | Linear | Linear | Linear |
| **Vaccine effectiveness  against infection** | 65% | 65% | 65% | 65% | 65% | 65% | 65% | None (while vaccine effectiveness against symptomatic infection exists) | 65% |
| **Vaccine effectiveness against hospitalization** | 83% | 66%-80% | 90% | 65% | Varying depending on past immune status | 65% | Varying depending on past immune status | 65% | 80-90% |
| **Waning of natural immunity** | Leaky, fitted | Leaky, assumed (gamma distributed with 3-6-month average time) | Leaky, fitted | Leaky, assumed (exponential with 6-month average time) | Leaky, assumed (gamma distributed with 2.5-4-month average time) | All-or-nothing, assumed (exponential with 8-month average time) | Leaky, assumed (exponential with 4-month average time) | Leaky, assume (exponential with 3-month median waning time) | All-or-nothing, assumed (exponential with 5-month average time) |
| **Waning of vaccine immunityinfection** | Leaky, fitted | Leaky, assumed (gamma distributed with 2-4-month average time) | Leaky, fitted | Leaky, assumed (exponential with 6-month average time) | Leaky, assumed (gamma distributed with 2.5-4-month average time) | All-or-nothing, assumed (exponential with 6-month average time) | Leaky, assumed (exponential with 4-month average time) | Leaky, assume (exponential with 6-month median waning time) | All-or-nothing, assumed (exponential with 5-month average time) |
| **Boosting effect from multiple infection** | None | None | None | None | None | None | None | None | None |
| **Immune escape** | Immune escape affects all protective effectiveness | Immune escape affects waning time of immunity | Immune escape only affects protective effectiveness against infection and symptomatic infection | Immune escape only affects protective effectiveness against symptomatic infection | Immune escape only affects protective effectiveness against infection | Immune escape affects all preexisted immunity | Immune escape affects all protective effectiveness | Immune escape affects all protective effectiveness | Immune escape only affects protective effectiveness against infection and symptomatic infection |
| **Seasonality** | Fitted with dynamics from 2020-2023 (varied by state) | Fitted with dynamics from 2020-2023 (varied by state) | Modeled with indoor contact rates  (varied by state) | Fitted with epidemics dynamics from 2020-2023 (varied by state) | Modeled with 2021 cubiq contact data (same across all states) | Modeled with date variation (same across all state) | Fitted with dynamics from 2020-2021 (same across all state) | Modeled as a Cosine function (same across all states) | Fitted with dynamics from 2020 - 2023 in North Carolina |
| **Peak timing of Seasonality (month)** | November-January | January | December | January-Feburary, depending on state | December- January (April-June depending on the scenario and state) | December- January (summer for children in school) | December- January | January | October-December |
| **Importations** | None | None | None | None | None | None | None | None | None |

**References**

1. flepiMoP. FLexible EPIdemic MOdeling Pipeline (flepiMoP). 1 Oct 2023 [cited 26 Jan 2024]. Available: https://iddynamics.gitbook.io/flepimop/

2. Davis JT, Chinazzi M, Perra N, Mu K, Pastore Y Piontti A, Ajelli M, et al. Cryptic transmission of SARS-CoV-2 and the first COVID-19 wave. Nature. 2021;600: 127–132. doi:10.1038/s41586-021-04130-w

3. España G, Cavany S, Oidtman R, Barbera C, Costello A, Lerch A, et al. Impacts of K-12 school reopening on the COVID-19 epidemic in Indiana, USA. Epidemics. 2021;37: 100487. doi:10.1016/j.epidem.2021.100487

4. Bouchnita A, Bi K, Fox S, Meyers LA. Projecting Omicron scenarios in the US while tracking population-level immunity. Epidemiology; 2023 Aug. doi:10.1101/2023.08.11.23293996

5. Patel MD, Rosenstrom E, Ivy JS, Mayorga ME, Keskinocak P, Boyce RM, et al. Association of Simulated COVID-19 Vaccination and Nonpharmaceutical Interventions With Infections, Hospitalizations, and Mortality. JAMA Netw Open. 2021;4: e2110782. doi:10.1001/jamanetworkopen.2021.10782
