# Supplementary material for: Potential impact of annual vaccination with reformulated COVID-19 vaccines: Lessons from the US COVID-19 scenario modeling hub
Source: PLoS Med. 2024 Apr 17;21(4):e1004387. doi: 10.1371/journal.pmed.1004387 (PMC11062554; doi:10.1371/journal.pmed.1004387)
Supplement: Attachment — Submitted filename: jung.pdf [file pmed.1004387.s010.pdf]

This paper presents ensemble models of the potential impact of COVID–19 in the United States.

I was asked for a statistical report and I interpret that to include all aspects of the design and conduct of the study.

Points of detail

Page 4 Is there any reason why one team only projected for North Carolina? Does it have some unusual constellation of risk factors for disease?

Page 4 What degree of trimming was applied in the pooling and was that a data–driven decision or was it based on theory?

Page 5 Do I understand the first caveat as meaning that the authors produced eight estimates and then combined them? I assume so and that make sense but, as written, it is not entirely clear to me so some expansion would be helpful.

Page 5 How exactly were the estimates combined in metafor? Using the threefold classification of Rice et al. (2018) which model did the authors assume: common effect, fixed effects, random effects? I would have thought that in this context fixed effects would be indicated.

Page 5 I am afraid the second caveat completely escaped me. Do the authors mean projection intervals here rather than prediction intervals? The sentence starting 'Hence' was also unclear to me.

Page 6 Is it reasonable to compare mortality rates for the scenarios where 65+ receive vaccination with the total population?

Page 6 Presumably by now the coverage for the 2023 campaign would be known so we could tell whether the rate is continuing to fall.

Page 7 Good to supply the code but I get a 404 error when I click on the GitHub link.

Figure 3 In the caption should the last sentence be split? I do not understand what '... at saturation considering the higher severity ...' is doing here.

Supplementary Figure S1 If this is an attempt to portray shaded regions of three colours superimposed then that is not a good look. If it is not then I do not understand it. If the authors want to show three regions then I think plotting the lines in a more saturated colour and then the boundaries of the regions faint and dotted or dashed might work.

Supplementary Figures SF2 and SF3 'lower/higher values'. Values of what? The colours seem almost redundant here but if they are carrying some message then should we not have a key to tell us what red, orange, gree and blue are numerically? Bear in mind too that people with a colour vision anomaly may find this hard to decipher.

Supplementary Figures SF4 and SF5 Here we do get a key but again I wonder whether the colour adds much.

Should we, somewhere, be told who the eight/nine teams were? Perhaps in the supplement or acknowledgments?

Point of more substance

Suppose the European Commission decided that it wanted to do a similar modelling exercise. After due process it could fund teams, perhaps in a range of member states, to replicate this methodology. My feeling is that there is insufficient detail in the methods here to enable that and we need much more. It can always go into the supplement to avoid breaking up the flow of the paper.

Summary

Quite a few points for clarification.

Michael Dewey

References

K Rice, J P T Higgins, and T Lumley. A re–evaluation of fixed effect(s) meta–analysis. *Journal of the Royal Statistical Society*, 181:205–227, 2018.
