# Supplementary material for: Potential impact of annual vaccination with reformulated COVID-19 vaccines: Lessons from the US COVID-19 scenario modeling hub
Source: PLoS Med. 2024 Apr 17;21(4):e1004387. doi: 10.1371/journal.pmed.1004387 (PMC11062554; doi:10.1371/journal.pmed.1004387)
Supplement: Attachment — Submitted filename: Point-by-point response.docx [file pmed.1004387.s011.docx]

***Response to Academic Editor:***

1. Please add line numbers to aid with reviewing.

>> We now include the page and line numbers.

2. Methods, Page 4: Clarify what the “… recommendation for eligible groups…” entails. I.e. which groups would be eligible for vaccination under this scenario.

>> To provide clarity regarding eligible groups for the annual vaccination with reformulated vaccines, the term ‘*all eligible groups*’ was replaced with ‘*all ages eligible for vaccination based on the US Food and Drug Administration (FDA) approval*’ in the revised manuscript.

[P5 L194-196] *“(1) no recommendation for annual vaccination with a reformulated vaccine, (2) a recommendation for those aged 65 and above (65+), and (3) a recommendation for all ages eligible for vaccination based on the US Food and Drug Administration (FDA) approval [16].”*

3. Methods/Results would benefit from a greater description (and comparison) of the models included in the ensemble (perhaps in the form of a table with supporting text?)

>> We appreciate the editor’s suggestion. We added a description of the contributing models in the *Supplementary Materials* (**Table S1**).

4. Seasonality: My understand from the limited description in the methods is that all modelling teams assumed some form of annual seasonality (presumably with a single winter peaks, given the predictions presented in Figure 1?). However, over 2023, many countries continued to see several peaks throughout the year, mostly unrelated to season. The authors should undertake sensitivity analysis, allowing greater flexibility in seasonality.

>> While teams were required to have some representation of seasonality, our scenarios allowed teams the flexibility to project future COVID-19 waves without imposing constraints on the timing and extent of seasonal forcing (e.g., not restricted to having a single seasonal peak). As shown in **Table S1**, each team adopted a distinct approach to incorporate seasonality into the model, considering various factors like environmental variables and human contact patterns, with calibration based on retrospective data. We make this point clear in the Method section now, stating as follow:

[P5 L215-217] *“Furthermore, the incorporation of SARS-CoV-2 seasonality was required, though teams had discretion in terms of its implementation without any constraints on the timing and extent of seasonal forcing (e.g., not restricted to having a single seasonal peak;* ***Table-S1****).”*

Furthermore, in addition to seasonality, the antigenic drift of SARS-CoV-2—one of the key axes in our scenarios—also impacts the timing of waves, which could potentially contribute to observed peaks during the summer season in 2023. It should be noted that in past rounds focused on variants, participating models have been able to recreate these off-season peaks.

While we agree that it would be good to understand more about what factors drive seasonal patterns and their impacts on projections, given the multi-team and real-time operational nature of Scenario Modeling Hub (SMH), it is not practical at this point to perform this retrospective analysis. Besides, SARS-CoV-2 has been circulating for less than 4 years, with much of past patterns driven by pandemic rather than epidemic behavior. As a result, a comprehensive assessment of seasonal drivers remains very tricky, compared to other respiratory diseases for which 10-30 years of stable seasonal records are available. A hub structure allows aggregation of results representing different assumptions about seasonality, which is particularly favorable in situations of high scientific uncertainty. We have listed this under the limitations.

[P8 L354-359] *“Second, the potential impact resulting from variations in the details of the modeling approach (e.g., seasonality) and parameter values, determined at the teams' discretion, were not quantified due to the multi-team and real-time operational nature of the SMH framework. A hub structure is particularly useful when there is valid scientific uncertainty about the role of specific drivers of disease dynamics, including seasonality.”*

5. Major (perhaps unjustified assumption) is that annual booster vaccination uptake will mirror uptake when boosters became first available in Sept 2021. US data shows that more recent uptake has been considerably lower, and varying by age group. My feeling is that future rounds of booster uptake will continue to be low. Thus, I am not convinced that this assumption holds and gives an overly optimistic outcome. The authors should revise analysis to include modelling of scenarios where booster uptake is lower - and in line with more recent US estimates - and discussion in greater detail the implications of this.

>> We acknowledge the editor’s point regarding the discrepancy between our scenarios (i.e., based on state- and age-specific coverage of the first booster dose) and the actual uptake of reformulated vaccines in fall-winter 2023-2024. However, the primary objective of our study is not to forecast future waves (what will happen), but rather to project the potential benefits of annual vaccination with reformulated vaccines under different scenarios (what would happen given the scenario). Such contrast between projected scenarios (i.e., bracketing the reality) is particularly useful to inform future public health planning. Therefore, the assumed coverage in our scenarios does not necessarily have to mirror the currently observed coverage. We further note that the modeled uptake assumptions were developed in discussion with the group of public health experts who would be ultimately using our results. In response to this comment, we explicitly addressed this discrepancy and its potential impacts on projected outcomes in the limitation section of the revised manuscript. We also included a supplementary comparison figure illustrating the assumed and observed vaccine coverages using the latest US CDC data (**Figure S6**).

[P8 L332-340] *“In the discrepancy period, assumptions of vaccine coverage matched well with realized uptake, suggesting that factors other than vaccine assumptions drove the difference between observed and projected disease dynamics. We note that later in the fall of 2023, the observed reformulated vaccine uptake saturated at a lower level than our all-age scenario (although vaccine coverage observations are well bracketed by our set of scenarios;* ***Figure-S6****). However, since our study primarily focuses on projecting the potential advantages of annual vaccination (rather than forecasting), our assumed uptake patterns in any individual scenario may not necessarily mirror the observed ones.”*

6. Wasn’t clear to me whether any models included “boosting” effects of repeated infection over the study period. This needs to be clearly described, perhaps in the additional table suggested above.

>> Teams were free to incorporate the impact of multiple infections based on their best scientific understanding of the situation as there are a myriad of ways this could be captured. To do so we have asked each team to provide a description of how multiple infections impact immunity, and whether immunity is all-or-nothing or “leaky” in the newly added **Table S1**.

7. Reader will want to see more detailed descriptions of the age-group specific effects under various scenarios (currently in a figure, but greater description in Results, and in Discussion section would be helpful).

>> We appreciate the editor’s comment. To support a variety of approaches and age structures, SMH has only requested aggregate numbers of hospitalizations and deaths to be reported from each team. While we recognized that having age-specific outcomes be reported could be a benefit to the users of SMH results, this has not been our policy over the 17 rounds of the project. Instead, our approach provides aggregate estimates of the benefits of targeting different age groups by design of the scenarios (rather than the outcomes). At this point it would be a challenge to obtain the age-specific hospitalizations and deaths from each team, as they may not have been logged given they were not part of the outcome. We have highlighted in the Discussion that this is a limitation.

[P8 L359-362] *“Third, to accommodate diverse modeling approaches, we focused on aggregated projections of hospitalizations and deaths across all age groups for each scenario, while the scenarios were designed with different age specific vaccine recommendations.”*

8. As we are now in early 2024, there is a whole year of empirical data (epidemiological, vaccine booster uptake) that could be compared to model predictions - currently only up to Aug 2023 - I think this whole-year comparison would be really important to show that model predictions have validity. If model predictions don’t align with 2023 epidemic trends, then the authors should interrogate why this is the case.

>> We have now updated **Figure 1 & Figure S1** to include empirical data up to December 15, 2023. The observed hospitalization and death trends generally correspond with our ensemble projections across all scenarios, while some discrepancy was noted in September–October 2023, primarily among southern states. We have also provided a comparison of our scenario assumptions and vaccine coverage data in Figure S6. We now comment on the comparison between scenario projections, scenario assumptions, and reality in the revised manuscript.

[P8 L330-332] *“Our ensemble projections in this scenario appear to align well with the empirically observed national-level hospitalizations, yet some discrepancy was noted in September–October 2023, primarily attributed to faster resurgences in southern states [14].”*

To further provide support for the validity of our hub approach, we now add a new quantile-quantile (QQ) plot (**Figure S7**) that compares the expected and actual coverage of each model and the ensemble. We focus on scenarios where boosters are recommended for all individuals (which corresponds to real-world practices) and study coverage for cumulative hospitalizations and deaths as of December 15, 2023. Across projected outcomes and scenarios, the ensemble projection demonstrates overall better performance compared to individual models and coverage values close to nominal levels, while most individual models exhibit either over- or underconfidence relative to the ensemble.

[P8 L340-343] *“Additionally, in scenarios with vaccination recommendations to all individuals, the ensemble outperforms individual models, wherein most show either over- or underconfidence relative to the ensemble (****Figure-S7****). Such improvement of the ensemble over individual models aligns with our earlier findings based on prior rounds of SMH projections [15].”*

9. A more philosophical question about the validity of combining estimates from multiple models using the ensemble approach: Some modelling scenarios are likely to be more plausible than others (more realistic assumptions, better quality input data etc), yet by combining models, do the authors assume that all model scenarios are given equal weight? I think readers will want to see much greater information on the range of estimates obtained from each individual model to allow judgement over whether the ensemble output estimates are plausible. Again, this could be in the form a a table/figure in the main results with 1-2 paragraphs added to describe.
>> As our primary objective is to assess the potential benefits of annual vaccination under varied scenarios, we have presented ensemble projections based on trajectories of individual models within each scenario, rather than combining projections across scenarios. That is, these projections were focused on *a priori* use for potential policy selections that might influence which scenario we were in. As mentioned above, we now compare actual vaccination to scenario assumptions in **Figure S6** and discuss evidence around immune escape assumptions in the discussion.

[P8 L324-340] *“Among six considered scenarios, the one with high immune escape (50% per year) and CDC vaccine recommendation for all age groups aligns most closely with real-world practices. The CDC advised vaccinating all individuals aged over 6 months on September 12, 2023 [31], and the predominant variant in December 2023 (Omicron EG.5.1) was suggested to have around 17% immune escape compared to the pre-existing variant in June 2023 (Omicron XBB.1.5). This is equivalent to an immune escape of around 40% per year, assuming the same transmissibility between two variants [32]. Our ensemble projections in this scenario appear to align well with the empirically observed national-level hospitalizations, yet some discrepancy was noted in September–October 2023, primarily attributed to faster resurgences in southern states [14]. In the discrepancy period, assumptions of vaccine coverage matched well with realized uptake, suggesting that factors other than vaccine assumptions drove the difference between observed and projected disease dynamics. We note that later in the fall of 2023, the observed reformulated vaccine uptake saturated at a lower level than our all-age scenario (although vaccine coverage observations are well bracketed by our set of scenarios;* ***Figure-S6****). However, since our study primarily focuses on projecting the potential advantages of annual vaccination (rather than forecasting), our assumed uptake patterns in any individual scenario may not necessarily mirror the observed ones.”*

A thorough evaluation of SMH results for this season would need to wait until late spring for complete empirical data to become available. Nevertheless, we have included a new analysis, showing an enhanced performance of ensemble projections in contrast to individual models across projection outcomes and scenarios (**Figure S7**). We also note that in previous work (<https://www.nature.com/articles/s41467-023-42680-x>), we showed consistently better performance of the ensemble model compared to individual models, which we reference in the manuscript.

[P8 L340-343] *“Additionally, in scenarios with vaccination recommendations to all individuals, the ensemble outperforms individual models, wherein most show either over- or underconfidence relative to the ensemble (****Figure-S7****). Such improvement of the ensemble over individual models aligns with our earlier findings based on prior rounds of SMH projections [15].”*

***Response to Reviewer 1:***

1. Could the authors develop on the impact on the results regarding the fact that the modelling teams didn't include non-pharmaceutical interventions in their models and that they were free to use or not the SARS-CoV-2 seasonality as a factor? In my opinion the seasonality could have a major impact on the two outcomes included in this review (eg. mortality and hospo choose whether totalization). Specially if SARS-CoV-2 will be circulating more at the same time as other viruses like influenza or RSV are. The accumulation of patients for multiple circulating seasonal viruses could overwhelm the health facilities and have an impact on mortality.

>> Given the limited implementation of non-pharmaceutical interventions during 2023 in the US, their future reactive changes have not been incorporated into projections. Additionally, while teams were required to include some representation of seasonality, our scenarios allowed teams the flexibility to project future COVID-19 waves without imposing constraints on the timing and extent of seasonal forcing (e.g., not restricted to a single seasonal peak). We make this point clear in the revised manuscript, and an overview of each team’s approach to incorporating seasonality into their model is provided in **Table S1**.

[P5 L215-219] *“Furthermore, the incorporation of SARS-CoV-2 seasonality was required, though teams had discretion in terms of its implementation without any constraints on the timing and extent of seasonal forcing (e.g., not restricted to having a single seasonal peak;* ***Table-S1****). Teams were directed not to consider changes in non-pharmaceutical interventions over the projection period, given their limited implementation in 2023.”*

We agree that the co-circulation of respiratory infectious diseases including influenza and RSV has the potential to influence projected outcomes, possibly through heightened burdens on healthcare facilities or changes in risk perception. Our Scenario Modeling Hub (SMH) has provided combined projections for the impact of influenza and COVID-19 since the start of the 2023-24 season, although we assume no interaction between these pathogens. These plots suggest that the combined impact of these pathogens in 2023-24 is likely to be lower than that in the preceding winter, so that surges are perhaps less probable than they were. We provided additional elaboration on this aspect in the limitation.

[P8 L349-354] *“Our scenarios also did not consider interactions with other infectious diseases, but they may impact our projections if there are significant changes in risk perception or healthcare burden during the co-circulation of respiratory infectious diseases (e.g., tripledemic in the 2022-23 season [34]). Nevertheless, projections of the combined impact of multiple pathogens for the 2023-24 season suggest a probable lower impact on the healthcare system compared to the prior season [35].”*

2. Regarding the target population for the vaccination, even though the authors mention that this is based on the CDC ACIP, in my opinion it would also be important to consider the immunocompromised individuals, specially given the fact that the uptake among this vulnerable group is quite low in the US (Tartof SY, Slezak JM, Puzniak L, et al. Analysis of mRNA COVID-19 Vaccine Uptake Among Immunocompromised Individuals in a Large US Health System. JAMA Netw Open. 2023;6(1):e2251833. doi:10.1001/jamanetworkopen.2022.51833)

>> We thank the reviewer for the comment. Teams were free to incorporate the additional demographic aspects (in addition to age structure) into their model. A brief description of the demographic aspects considered is now outlined in the newly added **Table S1**.

3. In my opinion is important to highlight in the limitations (although the authors partially do this when they refer to the "strict scenario assumptions") that there are many factor that can influence the number of hospitalizations and deaths apart from the vaccination and the antigenic drifts that were included in the models. I would put emphasis on this for potential readers who are not familiar with the topic.

>> We thank the reviewer for the comment. The potential assumptions in our scenarios that may influence the projected outcomes are now clarified in the limitation section.

[P8 L346-357] *“First and foremost, scenario projections are conditional on often strict scenario assumptions. Both vaccine coverage and effectiveness might deviate considerably from scenario assumptions, although historical trends of influenza vaccination suggest that achieving higher coverage is unlikely, especially in older populations [30,33]. Our scenarios also did not consider interactions with other infectious diseases, but they may impact our projections if there are significant changes in risk perception or healthcare burden during the co-circulation of respiratory infectious diseases (e.g., tripledemic in the 2022-23 season [34]). Nevertheless, projections of the combined impact of multiple pathogens for the 2023-24 season suggest a probable lower impact on the healthcare system compared to the prior season [35]. Second, the potential impact resulting from variations in the details of the modeling approach (e.g., seasonality) and parameter values, determined at the teams' discretion, were not quantified due to the multi-team and real-time operational nature of the SMH framework.”*

4. The link to the replication codes in GitHub gives a 404 error, meaning that the page doesn't exist. This should be corrected.

>> We apologize for the confusion. The URL of GitHub has been corrected in the revised manuscript ([*https://github.com/SungmokJung/covid19-scenario-modeling-hub-R17*](https://github.com/SungmokJung/covid19-scenario-modeling-hub-R17)*)*.

***Response to Reviewer 2:***

1. The common model parameters and assumptions used by all modeling teams are clearly laid out, but the rationale for the chosen values is lacking. A clear rationale is important since I expect the main results (hospitalizations and deaths averted) are sensitive to these parameter choices. In particular, the 65% effectiveness against symptomatic infection seems quite high given current knowledge of VE. Can the authors add some brief rationale for the parameter guidance given to all modeling teams?

>> We appreciate the reviewer's comment. In our scenario, we considered a 65% reduced risk of symptomatic infection after vaccination compared to the unvaccinated group. This should be understood as the risk reduction immediately after vaccination (i.e., before the waning of vaccine-induced protection) and for a perfectly matched vaccine (where the circulating variant is the variant included in the vaccine). This is a theoretical quantity as there is no comparable estimate available in the literature. Yet, we based our thinking on a prior study on the vaccine effectiveness (VE) of the bivalent mRNA vaccine (recommended by CDC on September 1, 2022). In this study, the VE against emergency department encounters/hospitalizations was estimated to be around 60% compared to unvaccinated individuals within 3 months of vaccination. We considered this value as a lower bound, considering the potential for waning and the impact of unreported previous exposures. In addition, given the difference in VE (against ED versus against symptomatic infection), we decided to set that against symptomatic disease as 65% to remain consistent with the definition of VE used in prior SMH rounds. We also ran this assumption by our CDC collaborators. We now cite the underlying study and provide clarity on the rationale behind our chosen value.

[P5 L205-210] *“Reformulated vaccines are presumed to have 65% vaccine effectiveness against symptomatic disease at the time of reformulation and immediately after receipt, with protection declining based on waning immunity and antigenic drift. This assumption was derived from a prior study showing a 60% vaccine effectiveness against emergency department encounters of the bivalent mRNA vaccine (fall 2022) [19], while considering potential underestimation due to immune waning and unreported previous SARS-CoV-2 antigen exposures.”*

2. Was any further guidance on effectiveness against severe outcomes (hospitalization and/or death) provided to the modeling teams? We have consistently seen that COVID-19 vaccines confer better, more durable protection against severe outcomes. Since the primary results focus on hospitalizations and deaths, not including the additional benefit from vaccination against severe outcomes may cause the number of hospitalizations/deaths averted to be underestimated.

>> We allowed each team to determine the vaccine effectiveness against severe outcomes at their discretion. All individual models incorporated an additional level of protection against severe outcomes compared to that against symptomatic infection. Details of these variations between the contributing teams are now outlined in the newly added **Table S1**, and it is also clarified in the revised manuscript.

[P5 L210-211] *“Vaccine effectiveness against severe outcomes was at the teams’ discretion based on their best insights (****Table-S1****).”*

3. I suggest clearly defining "antigenic drift" in the Introduction to clarify that the authors examine gradual genomic changes of SARS-CoV-2 rather than emergence of a substantially different variant. This will be particularly useful for when the authors later describe the high and low antigenic drift scenarios.

>> We thank the reviewer for the comment. We now clarify the definition of “antigenic drift” in the manuscript.

[P4 L139-141] *“Combined, seasonality and ongoing “antigenic drift (i.e., gradual genetic changes in a virus evading prior population immunity [8])” of SARS-CoV-2 make it highly likely that the virus will pose a persistent threat to public health for the foreseeable future.”*

4. For ease of understanding, I suggest providing additional information about what the two immune escape scenarios represent. Are these rates intended to solely inform changes in VE against symptomatic infection due to immune escape, or would this also affect SARS-CoV-2 virulence in some way?

>> In our scenarios, we assumed that an intrinsic virulence (i.e., probability of severe outcomes “given infection” in a completely naive population) would remain constant, while the immune escape would affect the probability of infection. However, the protective effectiveness of prior immune exposures against severe outcomes was left at the teams’ discretion based on their best insights into the situation, and the details of variations are now outlined in the newly added **Table S1**.

5. I'd be curious how the different models were consistent (or not) across the areas where teams were given flexibility. Is it possible to add a summary table to the Appendix comparing and contrasting key model decisions? This would provide additional context for the larger ensemble results.

>> In addressing both the reviewer's and the aforementioned editor's comments, we now incorporate brief descriptions of individual models in the *Supplementary Materials* (**Table S1)**.

6. A limitation mentioned in the Discussion is that all scenarios assumed continuous immune escape, but the detailed guidance to modeling teams on the referenced GitHub repository says that immune escape could be modeled continuously or as frequent new subvariants. Just to confirm, did all teams choose to model immune escape continuously? If not, was there any check to confirm that the immune escape represented by new subvariants matched the target antigenic drift?

>> We thank the reviewer for the comment. We have now clarified this section. All contributing teams had the flexibility to model immune escape, with explicit instructions for an annual rate of 20% and 50% in low and high immune escape scenarios, respectively. We now mention it in the revised manuscript. We have asked each team how they incorporated this aspect into their models, whether continuously or in stepwise occurrences in the newly added **Table S1**.

[P5 L188-190] *"The implementation of immune escape in their models was left at the discretion of teams (e.g., continuously or in stepwise occurrences;* ***Table-S1****) while ensuring that the annual levels align with the scenario definition."*

7. The final sentence of the Discussion reference to emergence of a completely new variant as "a significant level of antigenic drift within a very short span" but, at least based on influenza, this sounds like antigenic shift. Quite minor, but I suggest reframing as shift rather than drift.

>> We thank the reviewer for the comment. We replaced “*antigenic drift*” with “*antigenic change*” in the corresponding sentence.

[P59 L366-368] *“However, the emergence of new SARS-CoV-2 variants showing a significant level of antigenic change within a very short span (e.g., Omicron [35,36]) could increase the disease burden far beyond these projections.”*

***Response to Reviewer 3 (statistical report):***

1. Is there any reason why one team only projected for North Carolina? Does it have some unusual constellation of risk factors for disease?

>> The team exclusively projected outcomes for North Carolina based on their decision. To support a diverse range of approaches and models, Scenario Modeling Hub (SMH) has incorporated projections from all contributing teams, irrespective of the number of states they projected.

[P6 L223-226] *“Eight different modeling teams contributed projections of weekly incident and cumulative COVID-19 hospitalizations and deaths for April 15, 2023–April 15, 2025 for all states and at the national level (one additional team provided projections for only North Carolina based on their interest).”*

2. What degree of trimming was applied in the pooling and was that a data-driven decision or was it based on theory?

>> We thank the reviewer for the comment. In the process of ensembling trajectories from individual models, two outermost values were trimmed to reduce the variance of the aggregate, while assigning equal weight to all remaining values. Further elaboration on this aspect is now included in the limitations section, accompanied by a citation to our previous study, which provides comprehensive details on the applied ensemble approach (<https://www.nature.com/articles/s41467-023-42680-x>).

[P6 L229-230] *“Distributions at each week were combined using the trimmed-linear opinion pool method (LOP) to create ensemble projections (two outermost values were trimmed while assigning equal weight to all remaining values) [15,20–22].”*

3. Do I understand the first caveat as meaning that the authors produced eight estimates and then combined them? I assume so and that make sense but, as written, it is not entirely clear to me so some expansion would be helpful.

>> Yes, to estimate the expected impact of vaccination, we applied standard meta-analysis approaches based on the eight different expected impacts derived from each model. We have now provided a clear clarification of this point in the revised manuscript.

[P6 L236-242] *“Within each individual model, the expected impact of vaccination was determined by calculating the difference, or ratio, of projected deaths and hospitalizations between different vaccination scenarios sharing the same rate of immune escape, with variances estimated using the Delta method [23]. These individual model level estimates were then combined to produce an ensembled estimate of expected vaccine impact and associated confidence intervals (CIs) using standard meta-analysis techniques (with a random effects model) as implemented in the R package ‘metafor’ [24,25].”*

4. How exactly were the estimates combined in metafor? Using the threefold classification of Rice et al. (2018) which model did the authors assume: common effect, fixed effects, random effects? I would have thought that in this context fixed effects would be indicated.

>> Within the *metafor* package, we used a random effects model, and we clarified it in the revised manuscript as above mentioned.

5. I am afraid the second caveat completely escaped me. Do the authors mean projection intervals here rather than prediction intervals? The sentence starting `Hence' was also unclear to me.

>> To clarify that the object of SMH is projection, not forecasting, we intentionally use the term 'projection interval' in this manuscript. While retaining this term, we have clarified our objective (projection) in the revised manuscript.

[P5 L215-219] *“Furthermore, the incorporation of SARS-CoV-2 seasonality was required, though teams had discretion in terms of its implementation without any constraints on the timing and extent of seasonal forcing (e.g., not restricted to having a single seasonal peak;* ***Table-S1****). Teams were directed not to consider changes in non-pharmaceutical interventions over the projection period, given their limited implementation in 2023.”*

The meta-analysis approach (**Figure 2**) involves ensembling the vaccine impact estimates driven from each individual model, so that this outcome mathematically differs from assessing vaccine impacts from ensemble trajectories. We have provided further clarification on this distinction in the revised manuscript.

[P6 L246-247] *“Hence, vaccine impacts estimated from the meta-analysis are not directly reproducible by comparing ensemble projections for each scenario (which are not mathematically equivalent).”*

6. Is it reasonable to compare mortality rates for the scenarios where 65+ receive vaccination with the total population?

>> We are not clear on this concern. Given the concentration of mortality in the 65+ group, it has been a point of discussion as to whether much benefit would be obtained by vaccinating younger age groups. Comparing full population mortality between scenarios where only this group is recommended to vaccinate, versus a recommendation to vaccinate all groups, seems a reasonable way to explore this question.

7. Presumably by now the coverage for the 2023 campaign would be known so we could tell whether the rate is continuing to fall.

>> We appreciate the reviewer for the comment. In response to this and an earlier comment, we included a supplementary figure comparing our coverage assumptions with observations using the latest US CDC data (**Figure S6**). Importantly, there is a discrepancy between our scenario (i.e., uptake patterns of the first booster dose) and the actual uptake of reformulated vaccines. However, as mentioned earlier, our study's primary objective is not to forecast future waves (what will happen) but rather to project the potential benefits of annual vaccination with reformulated vaccines under different scenarios (what would happen given the scenario) to inform future public health planning. Hence, the assumed coverage in a particular scenario may not necessarily align with the currently observed coverage. Additionally, the modeled uptake assumptions were developed in consultation with public health authorities who would be utilizing our results. Further, it can be useful for the set of scenarios considered to bracket reality (which is the case here, see **Figure S6**). We explicitly addressed this discrepancy and its potential impacts on projected outcomes in the Discussion section.

[P8 L332-340] *“In the discrepancy period, assumptions of vaccine coverage matched well with realized uptake, suggesting that factors other than vaccine assumptions drove the difference between observed and projected disease dynamics. We note that later in the fall of 2023, the observed reformulated vaccine uptake saturated at a lower level than our all-age scenario (although vaccine coverage observations are well bracketed by our set of scenarios;* ***Figure-S6****). However, since our study primarily focuses on projecting the potential advantages of annual vaccination (rather than forecasting), our assumed uptake patterns in any individual scenario may not necessarily mirror the observed ones.”*

8. Good to supply the code but I get a 404 error when I click on the GitHub link.

>> We apologize for the confusion. The URL of GitHub has been corrected in the revised manuscript ([*https://github.com/SungmokJung/covid19-scenario-modeling-hub-R17*](https://github.com/SungmokJung/covid19-scenario-modeling-hub-R17)*)*.

9. In the caption should the last sentence be split? I do not understand what `. . . at saturation considering the higher severity . . . ' is doing here (Figure 3).

>> Due to the higher severity in the elderly, we deliberately chose the x-axis to represent vaccine coverage at saturation in the 65+ age group, rather than the entire population. To prevent any misinterpretation of the figure, we have explicitly stated the reason for this selection, and the legend of **Figure 3** is now modified as follows:

*“The x-axis represents the assumed vaccine coverage among 65+ at saturation considering the higher severity in 65+ (likely to have the most significant contribution to decreasing hospitalizations). Dots in each panel correspond to individual US states.”*

10. If this is an attempt to portray shaded regions of three colours superimposed then that is not a good look. If it is not then I do not understand it. If the authors want to show three regions then I think plotting the lines in a more saturated colour and then the boundaries of the regions faint and dotted or dashed might work (Figure S1).

>> To enhance the visualization of boundaries, we have now modified the projection intervals in the revised **Figure S1** by incorporating dashed lines.

11. `lower/higher values'. Values of what? The colours seem almost redundant here but if they are carrying some message then should we not have a key to tell us what red, orange, green and blue are numerically? Bear in mind too that people with a colour vision anomaly may fund this hard to decipher (Figure S2 & S3)

>> These figures present the peak hospitalizations per 100,000 (**Figure S2**) and peak timing (**Figure S3)** over the next two years in high immune escape scenarios, respectively. The color variation in these figures serves as a guide for readers to easily discern the order of US states in each value (either the size or timing of peak hospitalizations) by scenario and by season (2023–2024 and 2024–2025). **Figures S2 and S3** and their legends are now revised with color-blind-friendly colors.

*“The color variation denotes the order of US states in the peak COVID-19 hospitalizations by scenario and season. Shades of yellow indicate states with lower values and shades of blue indicate states with higher values.”*

*“The color variation denotes the order of US states in the peak timing of COVID-19 hospitalizations by scenario and season. Shades of blue indicate states with an earlier peak and shades of yellow indicate states with a later peak.”*

12. Here we do get a key but again I wonder whether the colour adds much (Figure S4 & S5).

>> We appreciate the reviewer's comment. **Figures S4 & S5** have been revised with color-blind-friendly colors. The color variations are now used to effectively visualize the order of US states in percent prevented COVID-19 outcomes (hospitalizations and deaths) by immune escape level and by different comparisons of vaccination scenarios.

*“The color variation denotes the order of US states in the percent prevented COVID-19 hospitalizations by scenario. Shades of yellow indicate states with lower values and shades of blue indicate states with higher values.”*

*“The color variation denotes the order of US states in the percent prevented COVID-19 deaths by scenario. Shades of yellow indicate states with lower values and shades of blue indicate states with higher values.”*

13. Should we, somewhere, be told who the eight/nine teams were? Perhaps in the supplement or acknowledgments?

>> We now mention contributing modeling teams and their team members in the acknowledgment.

14. Suppose the European Commission decided that it wanted to do a similar modelling exercise. After due process it could fund teams, perhaps in a range of member states, to replicate this methodology. My feeling is that there is insufficient detail in the methods here to enable that and we need much more. It can always go into the supplement to avoid breaking up the flow of the paper.

>> We now incorporate descriptions of individual models in the *Supplementary Materials* (**Table S1).** We note that the primary aim of this study is to project the potential impacts of annual vaccination using the ensembling projections from multiple teams, which is known to provide the most robust approaches for estimating the future burden of COVID-19, rather than delving into the specifics of each team's modeling approach. If a parallel effort were to be replicated in a different geographic region, it would be unlikely for participating teams to exactly replicate the models used here (in fact the EU Scenario modeling hub exists with a very similar goal, combining different models from those used here). Throughout the paper, we point the reader to additional papers from the Scenario Modeling Hub that address specific methodologies (particularly for the ensemble), and enhance the replicability of the hub approach.

***Response to Editorial Team:***

1. Please include line numbers in your revised manuscript, ideally not starting from 1 with each new page.

>> We now include the page and line numbers.

2. Please provide 95% CIs and p values for all results where appropriate (including the abstract), check and amend throughout. We suggest reporting statistical information in the following format: ‘x’; (95% CI [‘y’,’ z’] p value) and use commas as opposed to hyphens (as these can be confused with negative values) to separate upper and lower bounds.

For p values, please report as p<0.001 and where higher as 'p=0.002'. Please add the statistical method used to your method section. We also invite you to report p values to consistently to the third decimal digit - thousandths

>> The format of confidence intervals and projection intervals in the revised manuscript now follows the suggested one: x (95% CI [y, z]).

3. Of all authors who submit a modelling study we ask for inclusion of specific items, derived from Geoffrey P Garnett, Simon Cousens, Timothy B Hallett, Richard Steketee, Neff Walker. Mathematical models in the evaluation of health programmes. (2011) Lancet DOI:10.1016/S0140-6736(10)61505-X.

>> The primary aim of the study is to project the potential impacts of annual vaccination with reformulated vaccines via the ensembling projections from multiple teams, which is known to provide the most robust approaches for estimating the future burden of COVID-19, rather than delving into the specifics of each team's modeling approach. Hence, while we have not provided detailed information on each model to ensure replicability, we now incorporate brief descriptions of individual models in the *Supplementary Materials* (**Table S1).**

4. Thank you for providing all your data on <https://github.com/midas-network/covid19-scenario-modelinghub/>. However, reviewers had access issues - please double check if correct.

>> We apologize for the confusion. The URL of GitHub has been corrected in the revised manuscript.

5. Please structure your abstract using the PLOS Medicine headings: Background, Methods and Findings, Conclusions. Please remove all other subheaders.

>> We now have the structured abstract following the PLOS Medicine guidance.

6. At this stage, we ask that you include a short, non-technical Author Summary of your research to make findings accessible to a wide audience that includes both scientists and non-scientists. The Author Summary should immediately follow the Abstract in your revised manuscript. This text is subject to editorial change and should be distinct from the scientific abstract.

>> We now have the structured Author Summary in the revised manuscript.

7. Please remove all statements apart from acknowledgements, author contributions and abbreviations from the end of the main manuscript and include these only in the relevant parts of the manuscript submission form. Funding, competing interest, and data availability will be compiled as metadata.

>> We removed Funding, Data availability, and Competing interests.

8. Please present and organize the Discussion as follows: a short, clear summary of the article's findings; what the study adds to existing research and where and why the results may differ from previous research; strengths and limitations of the study; implications and next steps for research, clinical practice and/or public policy implications; followed by a one-paragraph conclusion. Please remove all subheadings within your Discussion e.g. Limitations and other considerations. We also like to ask you to broaden the discussion and reflect on what the results may mean outside the US.

>> We removed all subheadings in the Discussion section. In addition, although we believe that the quantified benefits of vaccination in our study can provide valuable insights to other countries outside the US, we opted to focus the discussion solely on the US setting given its potential variations driven by different epidemiological situations in other countries.
