# Supplementary material for: Potential impact of annual vaccination with reformulated COVID-19 vaccines: Lessons from the US COVID-19 scenario modeling hub
Source: PLoS Med. 2024 Apr 17;21(4):e1004387. doi: 10.1371/journal.pmed.1004387 (PMC11062554; doi:10.1371/journal.pmed.1004387)
Supplement: Attachment — Submitted filename: Point-by-point response.docx [file pmed.1004387.s012.docx]

***Response to Academic Editor:***

1. We are not convinced by the authors argument that this study is an exercise primarily in projecting the potential future benefits of vaccination. Assumptions for vaccine coverage that feed into projections have not matched the reality of what has happened. Similarly, (albeit to a lesser extent) the modelling input assumptions around seasonality have not borne out. Your main results are reported in terms of potential numbers of deaths and hospitalisations averted under these substantially higher vaccine coverage scenarios, but there is no scenario that reflects the reality of vaccine coverage and epidemic trajectory during 2023. As a result, we think you need to be clearer about this still, and be much more circumspect in the conclusions about the potential impact of COVID on hospitalisations and deaths, given the inputs haven't necessarily reflected reality.

>> We acknowledge the editor's concern regarding the discrepancy between our scenario and the real-world uptake of reformulated vaccines. In response to the editor's comment, we have included cautions regarding the interpretation of averted hospitalizations and deaths, highlighting differences between vaccine assumptions and observed coverage that may influence the realized benefits of vaccine interventions. However, we would like to emphasize that our analysis primarily focuses on projections (rather than forecasts), which aim to predict the likely course of the epidemic based on a particular scenario to help inform public health institutions and individuals before the actual vaccination campaign begins. In fact, our estimates were used by CDC/ACIP prior to the fall 2023 booster campaign to guide their recommendation decisions. We have explicitly clarified our objective in the revised manuscript.

Lastly, it is worth noting that while the assumed coverage in our scenario was higher than the actual uptake pattern at the national level, state-level coverage assumptions were comparable with observations in some states (e.g., DC, GA, NC), particularly among those aged 65 and over, who are likely to contribute significantly to COVID-19 hospitalizations and deaths. We have now added a new supplementary comparison figure, **Figure-S7**, illustrating state-level vaccine coverage using the latest US CDC data, and have also updated **Figure-S6** to reflect the most recent data.

[P8 L272-281] *“We note that later in the fall of 2023, the observed reformulated vaccine uptake saturated at a lower level than our all-age scenario (although vaccine coverage observations are well bracketed by our set of scenarios;* ***Figure-S6****). However, state-level uptake patterns were comparable with the range of scenario assumptions in some states (****Figure-S7****) [33], particularly among 65+, who are likely to have significant contributions to reducing severe outcomes. Of note, our study primarily focused on projecting the potential advantages of annual vaccination (predicting the likely course of the epidemic given the scenario, rather than forecasting) to inform public health authorizations before the actual vaccination campaign begins; hence, our assumed uptake patterns in any individual scenario may not necessarily mirror the observed ones. Nevertheless, caution should be exercised when interpreting our projected hospitalizations and deaths averted by annual vaccination, as these outcomes are likely somewhat overestimated due to such discrepancies in vaccine uptake patterns.”*

2. We were a bit disappointed you did not take up our suggestion to broaden the discussions to reflect on the possible global implication of your study, and where your study findings sit within a broader context. Given the readership of PLOS Medicine, it is important that you address questions about international implications of your research. Whilst we appreciate it would be inappropriate to make projections for other countries and regions, it would be of value to contrast the modelling here with efforts in other countries/regions.

>> We appreciate the editor’s comment and acknowledge the importance of discussing the global implications of our study within a broader context. In response to the editor's comment, we have integrated an additional paragraph into the revised manuscript. This paragraph specifically elaborates on the potential implications of our findings for countries beyond the US, along with potential variations that may arise due to differing epidemiological and demographic factors.

[P8 L287-294] *“Our ensemble projections have potential implications for countries beyond the United States, where regular re-vaccination serves as a key strategy against COVID-19. In light of this global relevance, our projections provide insight into the benefits of annual vaccination in mitigating the disease burden, along with related work conducted in the European context [34]. However, it is essential to note that the magnitude of impacts may vary across countries due to differing epidemiological and demographic factors. In particular, variations in age distribution, circulating variables, transmission dynamics, and time-varying immunity within each age group can substantially influence the impact of annual vaccination efforts.”*

3. Please address the remaining comment from reviewer #2 (see below)

>> We have addressed the comment from Reviewer 2 below.

4. Please move the Author Summary. This should be inserted directly after the Abstract.

>> We now move the Author Summary to follow the Abstract (P2 L35–P3 L67).

5. L 306: “is one of the most robust approaches for estimating COVID-19 … “ please rephrase as this claim is not validated. Suggest: “Despite its limitations, ensembling scenario-based projections from multiple teams is a useful tool for estimating COVID-19’s …” or something similar.

>> We have revised this section following the recommendation.

[P9 L325-327] *“Despite its limitations, ensembling scenario-based projections from multiple teams has proven to be useful for estimating COVID-19’s future burden and the potential benefits of vaccination [13,15], providing valuable information for public health planning.”*

6. Supplementary Materials: Please note that supplementary materials are not checked and will be posted as supplied by the authors. Therefore, please double check. Please cite your Supporting Information as outlined here: https://journals.plos.org/plosmedicine/s/supporting-information - Please note you may use almost any description as the item name of your supporting information as long as it contains an "S" and number. For example, “S1 Appendix” and “S2 Appendix,” “S1 Table” and “S2 Table", and ensure that all SI materials have a call-out (link) in your main text.

>> We have double-checked to ensure that all supplementary materials (including newly added **Figure-S7**) are labeled with "S" and referenced in the main manuscript.

***Response to Reviewer 2:***

1. The authors state that they guided teams to implement 65% vaccine effectiveness against symptomatic disease (lines 143-145). In Table S1, why is this described as 65% VE against infection (with only the UVA-EpiHiper team implementing 0% VE against infection and 65% VE against symptomatic disease)? This may seem like a minor quibble, but in the hierarchy of vaccine effects whether or not teams interpreted the guidance as VE against infection, regardless of symptoms, or as VE against symptomatic disease would impact their findings quite a bit. Park, Dushoff, Grenfell, and Weitz do a nice job exploring how varying levels of asymptomatic transmission impacts an epidemic's trajectory (doi:10.1093/pnasnexus/pgad106), which is analogous to how 65% VE against infection would avert a good deal of infections and asymptomatic transmission versus how a 65% VE against symptomatic disease would avert a lower proportion of infections and therefore have a larger infection count for conversion to symptoms/hospitalization. If this is a simple terminology issue (though the description of the UVA-EpiHiper team's choice implies otherwise), I suggest clarifying Table S1. Otherwise this should be acknowledged as a limitation of the work, since at this point teams should not be asked to rerun their models.

>> We appreciate the reviewer’s comment. We have verified that the UVA-EpiHiper team assumed no vaccine effectiveness against infection. Instead, the team assumed that vaccines protect individuals from symptomatic disease, with asymptomatic cases having lower infectiousness compared to symptomatic cases.

While the hub guided teams to set a 65% vaccine effectiveness against symptomatic disease, most teams assumed the same vaccine effectiveness against both symptomatic disease and infection, potentially underestimating the vaccine impact by overlooking its protective effectiveness against asymptomatic infections. We have now mentioned this point as a limitation in the revised manuscript.

[P9 L303-306] *“Additionally, for simplicity, most teams assumed equivalent vaccine effectiveness against infection and symptomatic disease, potentially underestimating the vaccine impacts by neglecting protection against asymptomatic infections [35].”*
